# Supplementary material for: Genome-Wide Identification and Expression Analysis of the Fructose-1,6-Bisphosphate Aldolase (FBA) Gene Family in Sweet Potato and Its Two Diploid Relatives
Source: Int J Mol Sci. 2025 Jul 30;26(15):7348. doi: 10.3390/ijms26157348 (PMC12347426; doi:10.3390/ijms26157348)
Supplement: Supplementary file 1 [file ijms-26-07348-s001.zip › ijms-3748872-supplementary.pdf]

Table S1. Identification of *FBAs* in *I. batatas*, *I. trifida*, and *I. triloba*.

	Gene Name	Gene ID	Gene ID in NCBI	Chr.	Gene Position
	IbFBA1	*g7510.t1*	CM008340.1	LG2	23986669-23989571
	IbFBA2	*g25147.t1*	CM008334.1	LG6	30937530-30939212
	IbFBA3	*g29379.t1*	CM008332.1	LG7	30029703-30032048
I. batatas	*IbFBA4*	*g45357.t1*	CM008339.1	LG11	28818530-28821067
	IbFBA5	*g53926.t1*	CM008333.1	LG13	20479353-20482055
	IbFBA6	*g58139.t1*	CM008335.1	LG14	2009045220092870
	IbFBA7	*g59775.t1*	CM008334.1	LG14	30380613-30382747
	ItfFBA1	*itf15g01090.t1*	CP025658.1	Chr15	565141-566893
	ItfFBA2	*itf09g13930.t1*	CP025652.1	Chr09	8609072-8611533
	ItfFBA3	*itf03g07070.t1*	CP025646.1	Chr03	4579402-4581767
I. trifida	*ItfFBA4*	*itf04g13320.t1*	CP025647.1	Chr04	10771864-10771864
	ItfFBA5	*itf02g17280.t1*	CP025645.1	Chr02	13622358-13624631
	ItfFBA6	*itf01g09380.t1*	CP025644.1	Chr01	8444376-8446903
	ItfFBA7	*itf09g00890.t1*	CP025652.1	Chr09	387510-390121
	ItbFBA1	*itb04g13840.t1*	NC_044919.1	Chr04	14016932-14021559
	ItbFBA2	*itb15g01150.t1*	NC_044930.1	Chr15	658966-660797
	ItbFBA3	*itb01g15870.t1*	NC_044916.1	Chr01	19156173-19158872
I. triloba	*ItbFBA4*	*itb09g14800.t1*	NC_044924.1	Chr09	10093867-10096731
	ItbFBA5	*itb03g07120.t1*	NC_044918.1	Chr03	5216685-5219001
	ItbFBA6	*itb02g14760.t1*	NC_044917.1	Chr02	10720808-10723397

Table S2. Primers used in this study.

Gene	Forward Primer	Reverse Primer	GC content (F/R)	Annealing Temperature (F/R)
actin	AGCAGCATGAAGATTAAGGTTGTAGCAC	TGGAAAATTAGAAGCACTTCCTGTGAAC	42.86%/39.29%	68.61℃/68.50℃
IbFBA1	GCTCAACTTCACGGATCAGC	GTAGGCCTGTCTGTTCGTCT	55.00%/55.00%	64.91℃/60.89℃
IbFBA2	ACCCTCTTGAAGCCCAACAT	AGCTTGTTCATGGCGTTCAG	50.00%/50.00%	64.63℃/65.24℃
IbFBA3	GCTTACACCCTCAACCTCCT	ATCCTGAGCTGCCTTCACAT	55.00%/50.00%	62.03℃/63.76℃
IbFBA4	GAACCAAGGGCGAGACTACT	AAGGATCTCGGGCTCAACAA	55.00%/50.00%	62.16℃/65.83℃
IbFBA5	AGCTCCTCTTCACATCACCC	GTCCCCTTGTCCACCTTGAT	55.00%/55.00%	63.23℃/65.03℃
IbFBA6	GAGACAAACAGGAGGGCTCT	CAGGAAGCTCAACAACACCC	55.00%/55.00%	62.36℃/64.62℃
IbFBA7	TGAGCTCGTTAAGACTGCCA	GACGTCGACCATCTTCTTGC	50.00%/55.00%	63.68℃/64.78℃

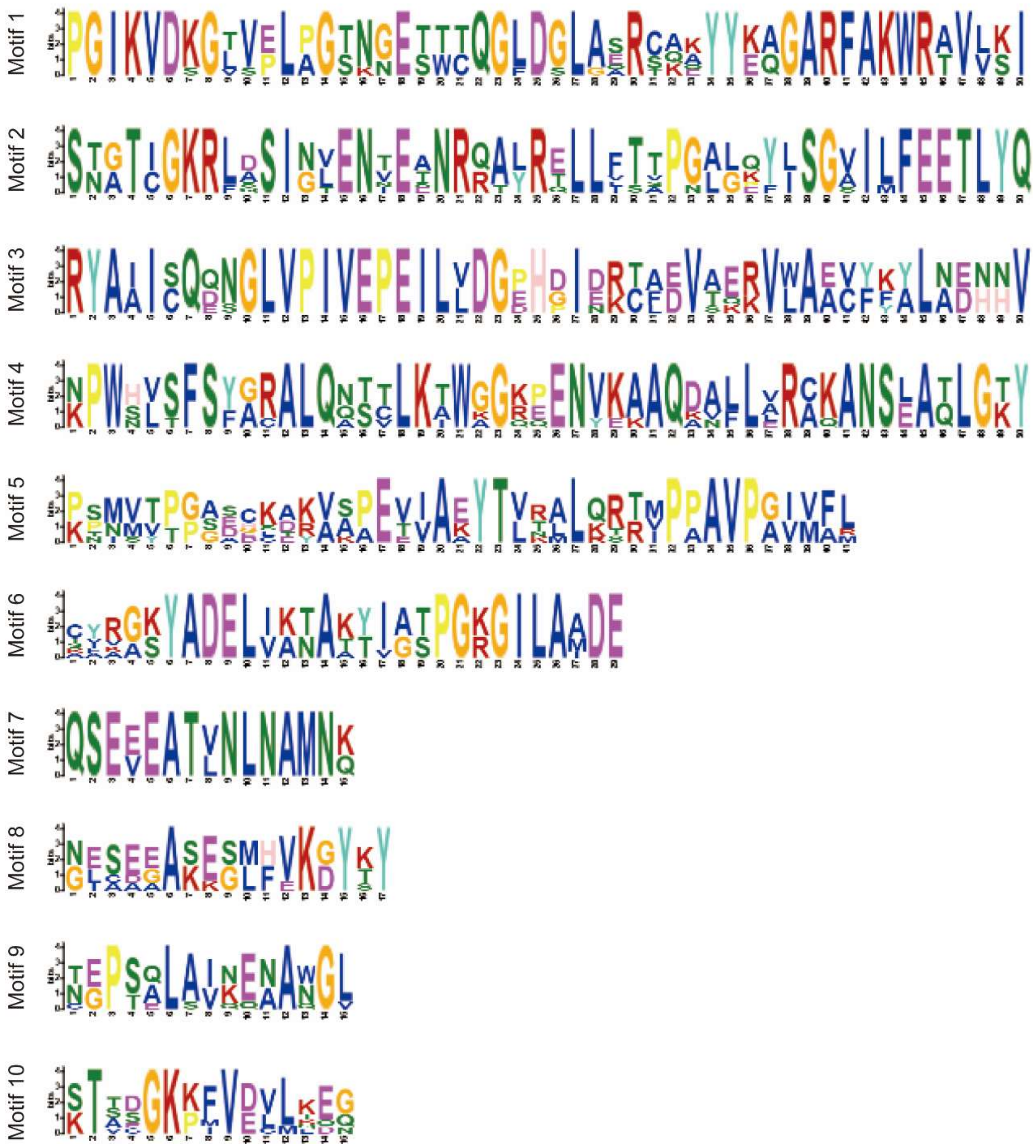

Figure S1. Sequence logos of the 10 conserved motifs.

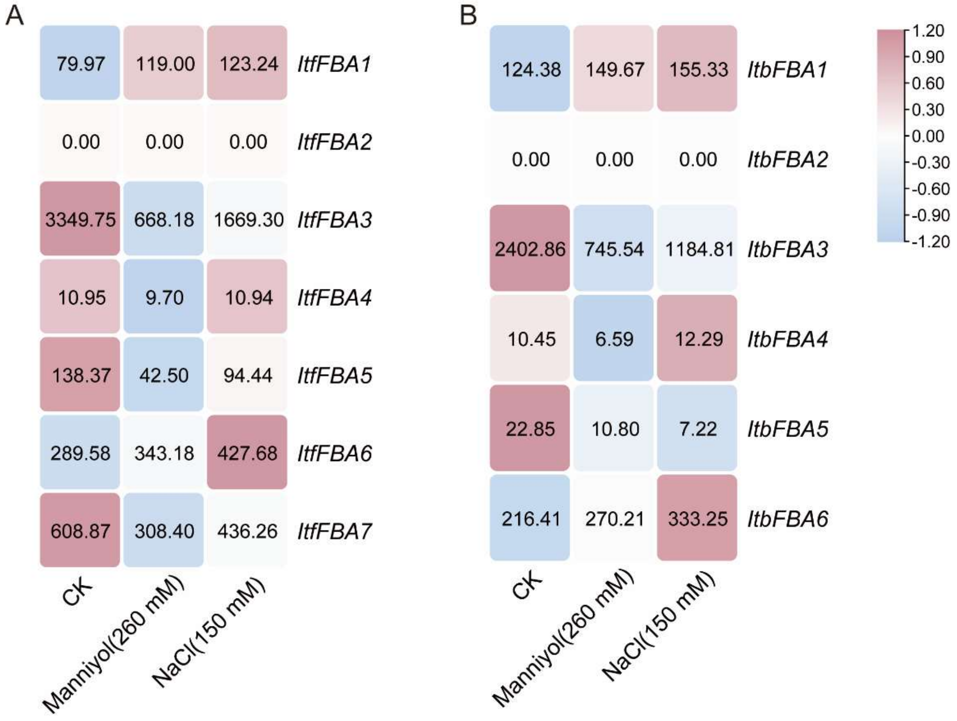

Figure S2. Expression analysis of *ItfFBAs* (**a**) and *ItbFBAs* (**b**) in response to drought and salt stresses as determined by RNA-seq. FPKM values are shown in the boxes.

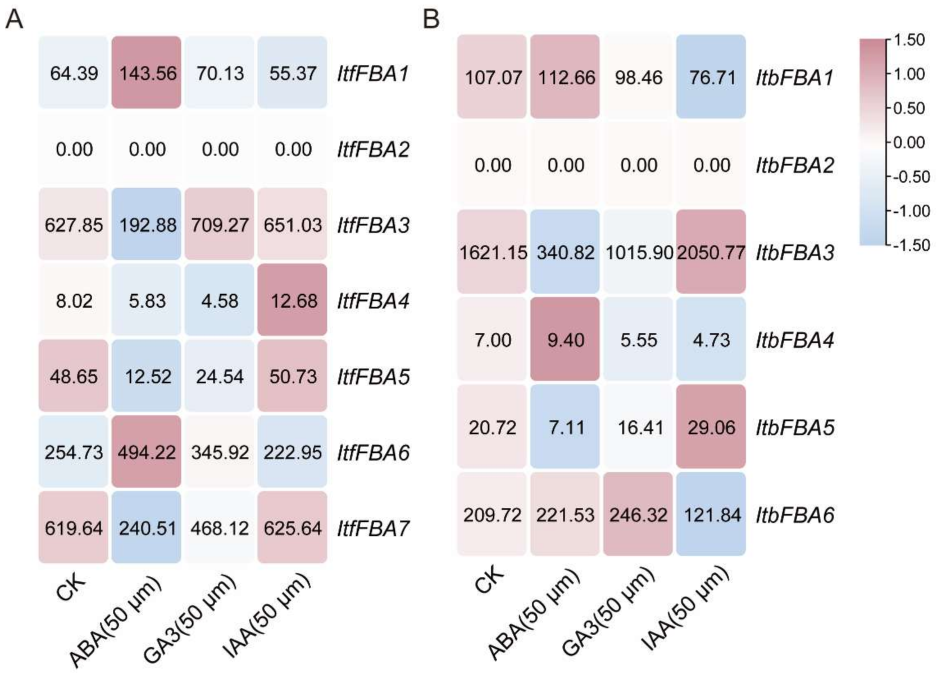

Figure S3. Expression analysis of *ItfFBAs* (**a**) and *ItbFBAs* (**b**) in response to different hormones (ABA, GA3, and IAA) as determined by RNA-seq. FPKM values are shown in the boxes.
